# A robust deep learning classifier for screening multiple retinal diseases on optical coherence tomography

**DOI:** 10.1038/s41598-025-19286-y

**Published:** 2025-10-09

**Authors:** Philippe Zhang, Gwenole Quellec, Sarah Matta, Laurent Borderie, Alexandre Le Guilcher, Tanguy Thiery, Béatrice Cochener, Mathieu Lamard

**Affiliations:** 1 LaTIM UMR 1101, Inserm, Brest, France; 2 University of Western Brittany, Brest, France; 3 Evolucare Technologies, Villers-Bretonneux, France; 4 Ophthalmology Department, CHRU Brest, Brest, France

**Keywords:** Retinal diseases, Machine learning, Data processing, Classification and taxonomy, Image processing

## Abstract

Retinal diseases are among the leading causes of visual impairment worldwide, where timely diagnosis and management are critical to prevent irreversible vision loss and blindness, especially in regions with limited access to ophthalmologists. While artificial intelligence (AI) has shown remarkable potential for screening ocular diseases, many existing models are heavily dependent on the datasets used for their development and often experience significant performance drops when tested on external datasets. These limitations in robustness and generalizability reduce their practical applicability in clinical settings. In this study, we proposed our newly developed deep learning architecture, FlexiVarViT is designed to enhance robustness and generalizability by addressing the unique characteristics of optical coherence tomography (OCT) imaging, such as variable data (e.g., number of slices, resolution), while processing B-scans at their native resolution (i.e., without resizing) to preserve fine anatomical details. Our method was evaluated on three datasets for the accurate detection and classification of multiple retinal pathologies using OCT images. These datasets represent diverse imaging devices (Spectralis and Optovue) and populations from France, Russia, and Iran. The results highlight the versatility, robustness, and generalizability of our model across various patient demographics and imaging systems, demonstrating significant improvements compared to state-of-the-art methods and emphasizing its potential for clinical application.

**Supplementary Information:**

The online version contains supplementary material available at 10.1038/s41598-025-19286-y.

## Introduction

According to 2023 data from the World Health Organization (WHO, https://www.who.int/), 2.2 billion people worldwide are affected by vision problems. Early and accurate diagnosis could help prevent irreversible vision loss for nearly one billion of them^1^. However, in many regions worldwide, a shortage of medical resources delays the diagnosis and treatment of these diseases^2^. Artificial intelligence has emerged as a promising, scalable, and real-time solution for the screening of retinal diseases, even in areas with limited medical resources^3^.

AI solutions based on Deep Learning (DL), have been extensively utilized to analyze various retinal imaging modalities^4,5^, such as color fundus photography^6–9^, ultra-widefield fundus imaging^10,11^, and optical coherence tomography (OCT)^12–14^, for detecting conditions including diabetic retinopathy (DR), age-related macular degeneration (AMD), diabetic macular edema (DME), glaucoma, myopia, and other retinal pathologies. These systems have demonstrated remarkable potential, automating disease detection with accuracy comparable to that of expert ophthalmologists^15,16^.

Among the many retinal imaging techniques, OCT stands out for its ability to generate 3D reconstructions of the retina, providing a more comprehensive view for disease screening^17^. Additionally, OCT delivers high-resolution, layer-specific information on retinal thickness, enabling more precise and detailed analysis. This makes it a highly valuable tool for integrating DL algorithms into clinical practice for early and accurate disease detection.

Several strategies have been developed for classifying volumetric OCT data. One widely used approach is Multiple Instance Learning (MIL)^18–20^, which involves classifying individual 2D slices and then aggregating slice-level predictions to form a single volume-level output, typically using mean or max pooling. MIL has been employed in disease detection, such as for AMD and DME in OCT volumes^21,22^. Another common approach is direct 3D volume classification^12,23^, which takes the entire volume as input to make a prediction. By processing the full volumetric data at once, the model effectively captures spatial relationships within the volume. This approach has been successfully applied to glaucoma detection using OCT^24,25^, with 3D ResNet architectures, showing its strength in leveraging spatial information.

More recently, the Variable Length Volume Feature Aggregation Transformer (VLFAT)^26^ has been introduced. This method aggregates the latent representations of each slice using a slice feature extractor and forms a comprehensive feature map for the entire volume using attention mechanisms^27^. This technique offers a balance between 2D slice-level and 3D volume-level approaches by processing slices independently while preserving inter-slice dependencies. VLFAT has shown robust performance in multi-class OCT classification tasks involving AMD, DME, and geographic atrophy (GA), which are primarily manifestations of AMD.

In addition, the foundation model RETFound^28^, based on the well-known Vision Transformer (ViT) architecture, represents a significant advancement in OCT analysis. Pretrained on large-scale OCT datasets, RETFound has demonstrated strong potential for generalization and robustness across diverse downstream tasks, including disease detection.

To address the clinical need for early detection of retinal diseases, we aim to develop a DL-based classifier capable of screening a wide range of pathologies, specifically focusing on AMD, DME, vitreomacular interface disease (VID), and a final category encompassing all other pathologies using OCT imaging. For AI to be effectively integrated into clinical practice, it must demonstrate both generalizability and robustness to adapt to real-world settings^5,6^. However, existing models often rely heavily on the datasets used during development and show significant performance drops when tested on external datasets. This challenge arises from variations in patient characteristics, imaging devices, and acquisition parameters, such as image resolution and slice count.

Furthermore, most current works focus on single-pathology detection^25,29,30^. This limits their ability to handle unseen diseases or multiple coexisting pathologies, which are frequent in clinical practice. A multi-disease framework is thus essential to improve diagnostic coverage and deliver reliable performance across diverse patient profiles.

In this study, we used three multi-pathology datasets—one private and two public—spanning different imaging devices and diverse demographic populations to ensure a comprehensive evaluation. Our model was trained on one dataset and tested on two others with distinct characteristics, enabling a robust assessment of its generalization capabilities. Our ultimate goal is to create a population- and device-independent tool that can be deployed across different countries and clinical environments, facilitating its widespread adoption in real-world practice.

## Methods

We utilized a total of four datasets in this study, comprising both private and publicly available datasets, with their detailed characteristics provided in Table 1. The private dataset (OCTBrest) focused on multi-disease classification, while the publicly available datasets (OCTDL, NEH, and Kermany) included both multi-disease and multi-lesion data.

**Table 1:** Characteristics of OCT datasets used in this study.

Characteristics	OCTBrest	OCTDL	NEH	Kermany
Role in model development	Training, cross-validation and test	Test	Test	Pretraining
Number of patients	251	821	148	5319
Age of patients (mean)	11 to 94 (64)	20 to 93 (63)	Not specified	Not specified
Sex ratio (Male-to-Female)	122:129	3:2	Not specified	Not specified
Number of volumes	663	NA	148	NA
Slices per volume (mean, SD)	14 to 62 (24, 11)	NA	19 to 61 (29, 15)	NA
Total slices	16,553	2,064	4,327	109,312
Number of classes	20	7	3	4
Imaging device	Heidelberg Spectralis	Optovue Avanti	Heidelberg Spectralis	Heidelberg Spectralis
Scan dimension, (mean) mm^2^	4.2 × 1.4 to 9.2 × 7.7 (6.0 × 4.8)	Not specified	8.9 × 7.4	Not specified
Azimuthal resolution, µm	4.9 to 13	Not specified	Not specified	Not specified
Lateral resolution, µm	4.9 to 13	15	Not specified	Not specified
Axial resolution, µm	3.9	5	3.5	Not specified
Slice resolution height, px	496	127 to 710	496, 512	496
Slice resolution width, px	512, 768, 1024, 1536	444 to 1270	496, 512	512, 768, 1024, 1536
Normal cases (count)	205	332	50	51,390
Pathological cases (count)	458	1,732	98	57,992
Pathologies included	See Table 2	AMD, DME, ERM, RAO, RVO, and VID	AMD and DME	DME, Drusen and CNV
Acquisition year(s)	2017-2018	2023	2017	2018
Population country	Brest, France	Yekaterinburg, Russia	Tehran, Iran	USA and China

Note: NA is indicated for OCTDL and Kermany as these datasets consist solely of slices and cannot be organized into volumes. Abbreviations AMD, age-related macular degeneration DME, diabetic macular edema ERM, epiretinal membrane RAO, retinal artery occlusion RVO, retinal vein occlusion, VID, vitreomacular interface disease.

The private OCTBrest dataset was collected and analyzed in accordance with the MR-004 reference methodology, established by the French CNIL (National Information Science and Liberties Commission). All experimental protocols were approved by the CNIL under this framework, which governs non-interventional research involving health data of public interest. For the three public datasets used in this study (OCTDL, NEH, and Kermany), all data were fully anonymized and complied with the ethical standards set forth by their original data providers. Informed consent was obtained from all subjects above 18 and from a parent or legal guardian for subjects under 18, in accordance with the ethical principles outlined in the Declaration of Helsinki.

### Datasets

The private OCTBrest dataset includes 663 OCT volumes from 251 patients (122 men and 129 women), aged 11 to 94 years, with an average age of 64 years (±16). These patients were seeking vision-related consultation at the Brest University Hospital in France between 2017 and 2018, using the Heidelberg Spectralis device and centered on the macula or the fovea. Each volume was annotated by one ophthalmologist expert in the retina (T.T., with 5 years of experience in retinal image analysis), identifying the presence of 20 different lesions or diseases (see Table 2). Normal eyes were classified based on the absence of any retinal lesions or diseases. To avoid memory bias, all right-eye scans were analyzed first, followed by the left-eye scans. The clinician had no access to the patients’ medical records during the annotation process. The images have scan dimensions ranging from 4.2 × 1.4 to 9.2 × 7.7 mm^2^ (average 6.0 × 4.8 mm^2^) with an axial resolution of 3.9 μm. The dataset also features azimuthal and lateral resolutions between 4.9 and 13 μm, with slice resolutions of 496 pixels in Height and varying widths of 512, 768, 1024, and 1536 pixels, ensuring high-quality retinal imaging.

**Table 2:** OCTBrest dataset - lesions and pathologies distribution.

**Lesions and pathologies**	**Number of patients/volumes (n=663)**
Drusen	208
Pigment epithelium abnormality	264
Serous retinal detachment	106
Pigment epithelial detachment	136
Subretinal hyperreflective exudation	49
Geographic atrophy	78
Cysts	200
Increased retinal thickness	197
Exudates	193
Epiretinal membrane	172
Macular hole	14
Vitreomacular traction	11
Other lesions	127
Age-related maculopathy	217
Exudative age-related macular degeneration	128
Atrophic age-related macular degeneration	49
Diabetic macular edema	35
Cystoid macular edema	56
Vitreomacular interface diseases	182
Other pathologies	98

Note: Patients may present with multiple pathological signs.

The public OCTDL (Optical Coherence Tomography Dataset for Image-Based Deep Learning Methods)^31^ dataset, obtained using the Optovue Avanti device and centered on the fovea, includes images from a Russian population. The dataset includes OCT scans from patients diagnosed with AMD, DME, Epiretinal Membrane (ERM), Retinal Artery Occlusion (RAO), Retinal Vein Occlusion (RVO), and VID, as well as normal cases. Specifically, there are 1231 scans for AMD from 421 patients, 147 scans for DME from 107 patients, 155 scans for ERM from 71 patients, 332 normal scans from 110 patients, 22 scans for RAO from 11 patients, 101 scans for RVO from 50 patients, and 76 scans for VID from 51 patients. In total, the dataset includes 2064 scans from 821 patients. The dataset labeling involved initial labeling by 7 trained medical students with consensus discussions, followed by review and consensus labeling by two clinical specialists, and final diagnosis confirmation by the head clinic expert.

The public NEH (Noor Eye Hospital)^32^ dataset contains 148 OCT volumes (comprising 48 AMD, 50 DME, and 50 normal subjects) acquired using the Heidelberg Spectralis device. The images have the same scan dimensions as OCTBrest (8.9 × 7.4 mm^2^, with an axial resolution of 3.5 μm). This dataset represents an Iranian population, offering additional geographic and demographic context. The images have slice resolutions of 496 pixels or 512 pixels in width and Height. A total of 4327 slices are available in the dataset, with each volume containing between 19 and 61 slices (an average of 29 slices per volume).

The public Kermany^14^ dataset, consisting of 109,312 OCT B-scan images, was developed to classify retinal conditions such as choroidal neovascularization (CNV), DME, drusen, and normal cases. Although it represents the largest dataset in terms of image volume, its primary focus is on multi-lesion classification rather than true multi-disease detection.

### Label standardization

**Fig 1:**
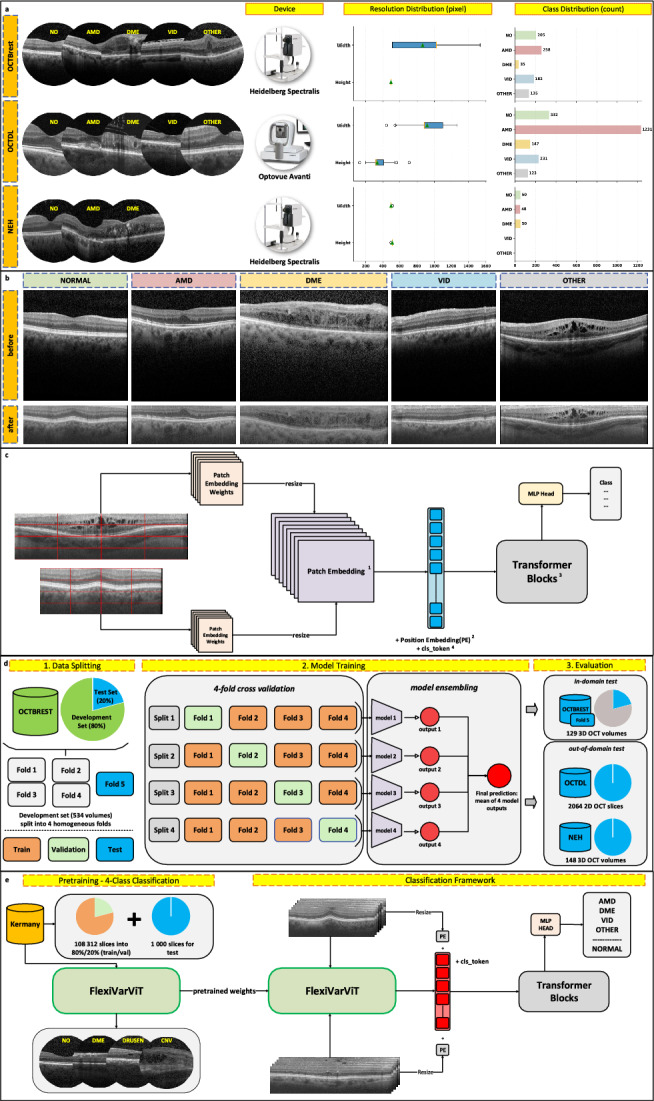
Complete study overview. **A** OCT Multi-Diseases Dataset. Among the three datasets, only OCTBrest is a multi-disease OCT dataset that includes patients with multiple co-occurring pathological signs. Among the 458 pathological OCT volumes, 122 presented with two pathologies, 12 with three, and 2 with all four. **B**Data Preprocessing using OCTIP on OCTBrest c FlexiVarViT Architecture d Model Development and Evaluation Pipeline e Our Final Classifier^1^.The patch embedding weights are dynamically resized to create variable-sized patches, enabling flexible handling of different image resolutions while preserving critical image details^2^. The positional encoding (PE) introduces spatial information, capturing the relative positions of slices within the volume, ensuring a coherent spatial representation throughout processing^3^. The Transformer Blocks processes the sequence of embedded patches by leveraging both spatial (through position embeddings) and contextual relationships via self-attention mechanisms, thus enhancing feature extraction and representation learning^4^. A classification token (cls_token) serves as a summary of the entire sequence and is passed through the MLP Head, which performs the final classification, predicting pathology categories based on the learned features.

The OCTBrest dataset consists of 20 classes, including 13 lesion types and 7 pathologies. The OCTDL dataset contains 8 distinct classes, while the NEH dataset comprises 3 classes. A senior ophthalmologist standardized the labels for the OCTBrest and OCTDL datasets into 5 main classes: Normal, AMD, DME, VID, and OTHER. In contrast, the original classes were retained for the NEH dataset. The class distributions (illustrated in Figure 1a) are as follows: the OCTBrest dataset contains 205 Normal cases, 258 cases of AMD, 35 cases of DME, 182 cases of VID, and 135 cases in the Other category. The OCTDL dataset includes 332 Normal cases, 1231 cases of AMD, 147 cases of DME, 231 cases of VID, and 123 cases classified as Other. Lastly, the NEH dataset comprises 50 Normal cases, 48 cases of AMD, and 50 cases of DME.

### Data preprocessing

We used the optical coherence tomography image preprocessing (OCTIP; https://github.com/leto-atreides-2/octip), developed by our laboratory LaTIM, which segments retinal layers and extracts only the essential parts of the image. OCT slices often contain significant noise and irrelevant information; this method effectively isolates the retinal layers, then flattens and aligns the images in depth, helping to standardize variations caused by different acquisition angles(see Figure 1b). The window Height after processing is set at 200 pixels^33^, a value recently validated on the large public dataset MARIO^34^. This size is sufficient to fully include all retinal layers, even in the most challenging cases such as severe edema or epiretinal membranes.

### Deep learning approaches

Three distinct approaches for classifying volumetric data were employed in this study: MIL, direct 3D volume classification, and the VLFAT. For the MIL approach, we employed RETFound^28^, a foundation model, pretrained on 736,000 OCT images with masked autoencoding. We applied both mean (RETFound-MIL-AVG) and maximum (RETFound-MIL-MAX) prediction pooling. For direct 3D volume classification, we employed MedNet3D^35^, a 3D foundation model based on ResNet^36^ architectures; among these, we utilized ResNet-18 (MedNet3D-R18) and ResNet-50 (MedNet3D-R50). MedNet3D models were pretrained on a wide range of datasets derived from various medical challenges, covering diverse imaging modalities, anatomical targets, and pathological conditions. Finally, for the VLFAT^26^ approach, we tested RETFound (RETFound+VLFAT) and a basic ViT^27^ pretrained on ImageNet (VLFAT) as a feature extractor. All our tested approaches support full-volume processing without subsampling the number of slices, ensuring the preservation of all available information. However, these methods require resizing OCT slices to a fixed size of 224x224 pixels due to architectural constraints, such as input size limitations during pretraining. This resizing process limits the ability to fully utilize the variable and high-resolution details inherent in OCT imaging, reduces image quality, and may negatively impact classification performance. To overcome these limitations, we proposed a novel deep learning architecture, FlexiVarViT, specifically designed to process variable-resolution data effectively while preserving critical image details. The proposed architecture is illustrated in Figure 1c.

FlexiVarViT is based on the FlexiViT^37^ architecture and is specifically designed to handle variable-high-resolution data without resizing, preserving the quality of high resolution images such as OCT images. Like FlexiViT, FlexiVarViT utilizes variable-sized patches; however, our model allows for handling data with variable resolutions by dynamically adjusting the patch sizes according to the image dimensions. In FlexiVarViT, we dynamically adjust the patch sizes to maintain a fixed number of patches per slice. This strategy ensures the preservation of a trainable position encoding without requiring additional modifications^27^. By keeping a constant number of patches, the position encoding can be applied consistently and continuously across all slices, ensuring that positional information is preserved and effectively utilized by the model. With FlexiVarViT, all images from the different datasets were processed at their original resolution following our preprocessing.

### Model development and evaluation across different parameters

We developed and evaluated our model through a systematic process aimed at ensuring both robustness and generalizability. Figure 1d illustrates the overall development and evaluation pipeline. The OCTBrest dataset was split into five folds. The distributions of the classes per fold are reported in Supplementary Table S1. A 4-fold cross-validation was performed on folds 1 to 4, producing four models. Model ensembling, utilizing averaged predictions, was employed to achieve robust performance. Final evaluation was conducted on fold 5, held as an independent test set. The OCTDL and NEH datasets were used solely for testing, allowing us to assess the model’s generalization to unseen data, including variations in imaging resolution and slice count.

### Pretraining strategies

We explored and compared two OCT-specific pretraining strategies: RETFound, based on masked autoencoding over 736,000 OCT B-scans, and supervised training on the Kermany dataset, the largest publicly available OCT slices dataset. First, we integrated RETFound pretrained weights into our FlexiVarViT architecture and combined them with the VLFAT framework (FlexiVarViT(retfound-p)+VLFAT), allowing our model to leverage large-scale OCT-derived features and potentially improve robustness and generalization.

However, both RETFound and ImageNet-based models are trained on downsampled images (224×224), which may limit their effectiveness in architectures designed to handle native-resolution OCT data.

To address this limitation, we leveraged the high-resolution capabilities of FlexiVarViT and applied supervised pretraining on the Kermany dataset (FlexiVarViT(kermany-p)). This dataset comprises 108,312 high-resolution slices from 4,686 patients, split into 80% for training and 20% for validation, with an additional 250 samples per class (from 663 patients) Held out for testing. FlexiVarViT achieved a mean one-vs-all AUC of 0.99 across the four classes on the test set, demonstrating excellent performance and potential for automated OCT diagnostics.

### Implementation details

All models were coded in PyTorch(v.2.4.0) and Python(v.3.11.9), and trained using the AdamW optimizer with a cosine scheduler with warmup and a learning rate of 6e-6 over 100 epochs on 4x NVIDIA A6000 GPU (48GB). All models were trained with the same seed for consistency. During training, we applied random slice selection following a Gaussian distribution^26^, with a random number of slices N chosen from the set {5, 10, 15, 20, 25}. For 3D-CNN models and FlexiVarViT, gradient backpropagation was performed over 8 iterations (batch size of 8) to handle variable-length data efficiently. The best model was selected based on the highest mean one-vs-all AUC (Area Under the receiver operating characteristic Curve) obtained on the validation set.

### Statistical analysis

The performance of each algorithm was assessed by computing one-vs-all AUC values for each class in each dataset, using the ‘sklearn’ package (v.1.5.1).

Statistical analyses were conducted in R (v4.4.0). We applied the Wilcoxon signed-rank test to compare two different algorithms, i.e. to compare two 13-tuples of AUC values.

## Results

The classification performance of the different approaches is summarized in Table 3 and Table 4. Table 3 presents global metrics including mean one-vs-all AUC, F1-score, sensitivity, and specificity. Table 4 details class-wise AUCs for each pathology across all datasets.

**Table 3:** Overall classification performance across the three multi-disease datasets.

	**OCTBrest**				**OCTDL**				**NEH**			
	mAUC	mF1	mSen	mSpe	mAUC	mF1	mSen	mSpe	mAUC	mF1	mSen	mSpe
MIL-RETFound+MAX	0.9150	0.6593	0.8815	0.6295	0.8410	0.4984	0.4915	0.9312	0.9530	0.6658	0.8067	0.5233
MIL-RETFound+AVG	0.9049	0.7003	0.6079	0.9495	0.8410	0.4984	0.4915	0.9312	0.9368	0.5581	0.5731	0.8600
MedNet3D-R18	0.8130	0.5869	0.5579	0.9168	0.5601	0.2228	0.4000	0.5992	0.8710	0.6472	0.6608	0.8399
MedNet3D-R50	0.8278	0.5895	0.4997	0.9570	0.5701	0.2136	0.3584	0.6352	0.9024	0.6817	0.6218	0.8900
RETFound+ VLFAT	0.9169	0.7787	**0.7919**	0.9102	0.8428	0.5531	0.6712	0.8047	0.9698	0.7332	0.7624	0.7533
VLFAT	0.9359	**0.8069**	0.7515	0.9582	0.8662	0.5486	0.6635	0.8643	0.9783	0.8274	0.8051	0.9367
FlexivarViT+ VLFAT	0.9541	0.7868	0.7798	0.9317	0.8964	0.5857	0.6406	0.9127	0.9870	**0.9118**	**0.8850**	0.9633
FlexivVarViT(retfound-p)+ VLFAT	0.9348	0.7440	0.7315	0.9218	0.8701	0.5516	0.6007	0.9049	0.9408	0.7608	0.6858	0.9567
FlexivVarViT(kermany-p)+ VLFAT **(ours)**	**0.9630**	0.7870	0.7577	**0.9590**	**0.9162**	**0.6514**	**0.7072**	**0.9210**	**0.9962**	0.8897	0.8494	**0.9767**

mAUC: mean one-vs-all AUC; mF1: mean F1-score; mSen: mean Sensitivity; mSpe: mean Specificity. Best scores are shown in bold.

**Table 4:** Class-wise classification performance (one-vs-all AUC) across the three multi-disease datasets.

	**OCTBrest**					**OCTDL**					**NEH**		
	AMD	DME	VID	OTHER	NO	AMD	DME	VID	OTHER	NO	AMD	DME	NO
MIL-RETFound+MAX	0.9268	0.9404	0.9387	0.8357	0.9331	0.8830	0.7368	0.7929	0.8412	0.9512	0.9275	0.9590	0.9727
MIL-RETFound+AVG	0.9150	0.9160	0.9361	0.8404	0.9171	0.8830	0.7368	0.7929	0.8412	0.9513	0.8938	0.9706	0.9461
MedNet3D-R18	0.8207	0.8482	0.9612	0.5589	0.8758	0.5613	0.5450	0.5238	0.6030	0.5672	0.8535	0.8110	0.9486
MedNet3D-R50	0.8418	0.8889	0.9542	0.5478	0.9062	0.5718	0.6058	0.4928	0.4871	0.6931	0.8385	0.9510	0.9176
RETFound+VLFAT	0.9375	0.8509	0.9625	0.8593	0.9744	0.8986	0.6924	0.8033	**0.8804**	0.9392	0.9708	0.9688	0.9698
VLFAT	0.9338	0.9634	0.9481	0.8700	0.9640	0.9074	0.8198	0.8866	0.7621	0.9549	0.9604	0.9824	0.9920
FlexivarViT+VLFAT	0.9667	0.9661	0.9631	0.9077	0.9666	0.9333	0.8839	**0.8934**	0.7996	0.9720	0.9746	0.9924	0.9941
FlexivVarViT(retfound-p) +VLFAT	0.9315	0.9648	0.9563	0.8562	0.9652	0.9018	0.8618	0.7728	0.8558	0.9582	0.8875	0.9637	0.9712
FlexivVarViT(kermany-p) +VLFAT **(ours)**	**0.9884**	**0.9715**	**0.9636**	**0.9098**	**0.9817**	**0.9743**	**0.8994**	0.8912	0.8295	**0.9865**	**0.9925**	**0.9982**	**0.9980**

The best performance for each class within each dataset is highlighted in bold.

Among all methods, FlexiVarViT pretrained on Kermany and combined with VLFAT achieved the best results across both in-domain and out-of-domain datasets, with mean AUCs of 0.963 on OCTBrest, 0.916 on OCTDL, and 0.996 on NEH. At the class level, it obtained the highest AUC in 11 out of 13 categories, confirming its effectiveness for multi-disease detection and its strong generalization capability.

VLFAT-based models consistently outperformed both MIL and 3D-CNN approaches. RETFound-MIL (using AVG and MAX pooling) achieved stable AUCs across datasets, ranging from 0.840 to 0.953. However, their F1-scores and sensitivities were lower, particularly on OCTDL, where the F1-score dropped to around 0.491 and sensitivity remained below 0.5. On OCTBrest and NEH, their F1-scores were more stable, around 0.659 to 0.703. No notable difference in performance was observed between the AVG and MAX groups.

3D-CNN models (MedNet3D-R18 and R50) showed the weakest performance overall. On OCTDL, both models reported low AUCs (approximately 0.560 to 0.570) and F1-scores (approximately 0.214 to 0.223). While they performed well on the VID class in OCTBrest (AUC = 0.954 to 0.961), performance declined sharply on OCTDL (AUC = 0.481 to 0.603), indicating poor robustness to domain shifts. MedNet3D-R50 slightly outperformed R18, but the gains were limited.

At the architectural level, performance differences between RETFound+VLFAT, ViT+VLFAT, and FlexiVarViT+VLFAT were minimal on OCTBrest. However, in out-of-domain evaluations with OCTDL and NEH, FlexiVarViT+VLFAT outperformed other methods in both AUC and F1-score.

Integrating RETFound weights into FlexiVarViT led to a slight performance drop across most metrics and classes. In contrast, supervised pretraining on the Kermany dataset consistently improved performance, with AUC gains in 12 out of 13 classes and a 10% increase in F1-score on OCTDL. On NEH, a small decrease in F1-score (from 0.912 to 0.890) and sensitivity (from 0.8850 to 0.8494).

Interestingly, for the OTHER category in OCTDL, the RETFound-based model achieved the top AUC score of 0.880, outperforming all other models for this specific class.

**Fig 2:**
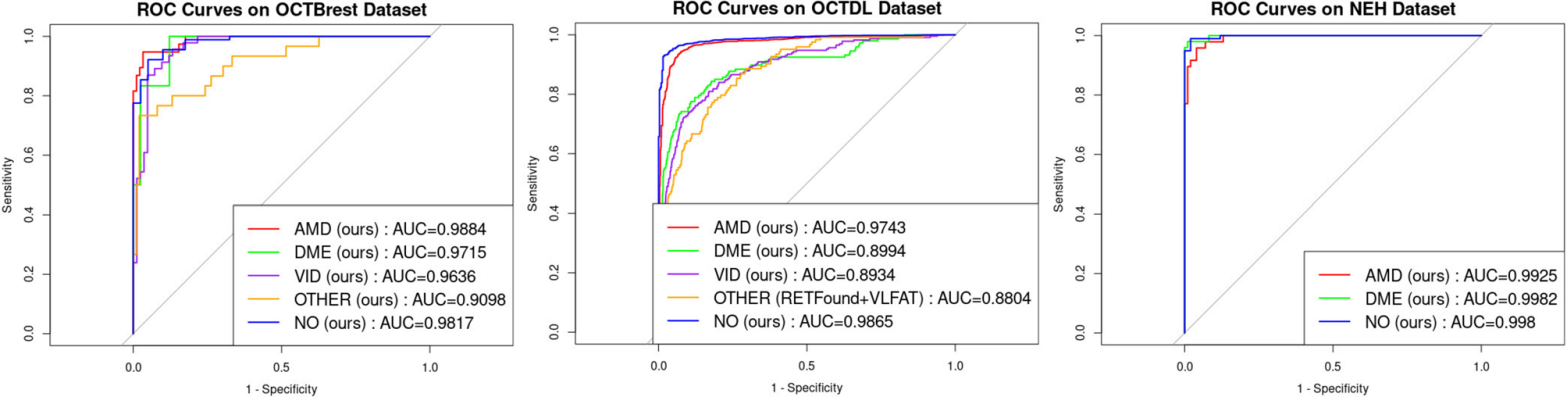
Best ROC Curves per dataset.

Figure 2 presents the ROC curves illustrating the best performances for each class and dataset. The computational costs of our algorithms on the test sets of the three datasets are reported in Table 5. In addition, the detailed confusion matrices for each method and dataset are provided in the supplementary Figure S1.

**Table 5:** Number of parameters and inference time for OCT classification models.

	Backbone	Parameters	OCTBrest	OCTDL	NEH
RETFound-MIL(AVG/MAX)	ViT-L	303M	8.26± 19.62	8.48± 0.25	8.7± 12.11
MedNeT3D-R18	3D ResNet18	33M	0.85± 3.1	1.04± 0.18	0.85± 5.24
MedNeT3D-R50	3D ResNet50	46M	1.75± 9.01	1.67± 0.14	1.85± 10.94
RETFound+VLFAT	ViT-L	432M	6.52± 4.76	5.98± 0.5	6.62± 3.31
VLFAT	ViT	164M	4.52± 2.5	4.67± 0.28	4.58± 3.33
FlexiVarViT+VLFAT	FlexiVarViT	164M	5.04± 8.42	4.35± 1.74	4.73± 3.06
FlexiVarViT(kermany-p)+VLFAT					
FlexiVarViT(retfound-p)+VLFAT	FlexiVarViT-L	433M	6.69± 5.19	6.28± 2.15	6.9± 4.86

Note: Values correspond to inference time in milliseconds (median ± standard deviation), measured on an NVIDIA A6000 GPU (48GB) with a batch size of 1.

## Statistical analyses

**Fig 3:**
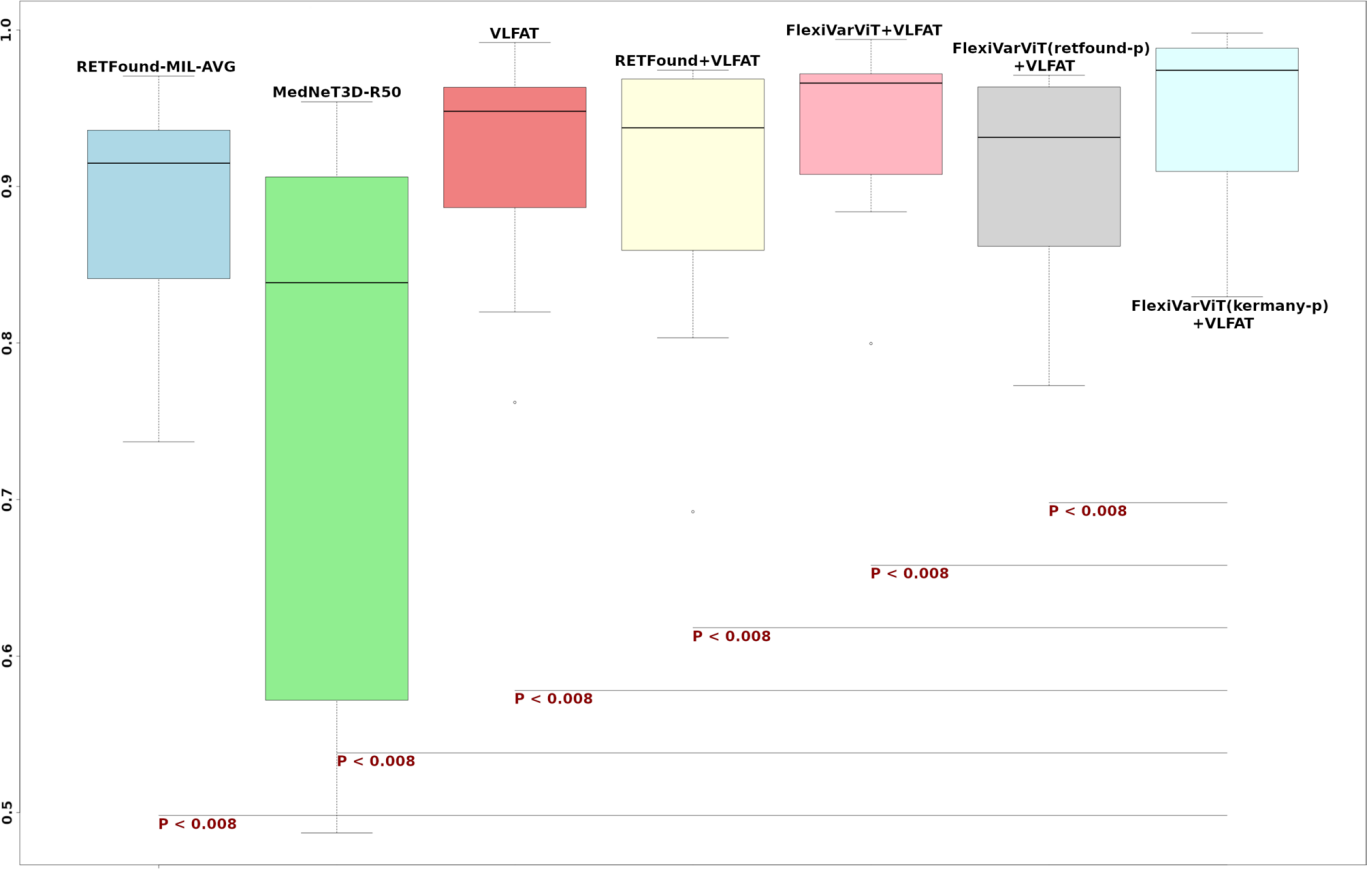
Wilcoxon signed-rank test – AUC. Note : Due to the minimal differences between RETFound-MIL-AVG and RETFound-MIL-MAX, as well as MedNet3D-R18 and MedNet3D-R50, we selected the best-performing model of each pair based on the average AUC across the 13 classes.

Figure 3 shows boxplots of the 13 AUC scores obtained across the three datasets for each model. Due to the similar performance observed between RETFound-MIL-AVG and RETFound-MIL-MAX, and between MedNet3D-R18 and MedNet3D-R50, only the best-performing model (based on the average of the 13 AUC) was retained for statistical comparison.

Each of these models was compared to our final model : FlexiVarViT(kermany-p)+VLFAT. This model achieved the highest median AUC and exhibited a narrow interquartile range, reflecting both accuracy and stability across datasets.

Bonferroni correction was applied to set a conservative significance threshold of 0.008 (0.05/6). All pairwise comparisons resulted in p-values below this threshold, confirming that FlexiVarViT (kermany-p) + VLFAT is statistically significantly superior to all other tested methods.

## Discussion

This study evaluated the performance of various deep learning strategies for multi-disease classification using OCT images. To ensure robustness and generalizability across diverse patient populations and imaging devices, key factors for clinical integration, models were tested on three distinct multi-disease datasets: OCTBrest, OCTDL, and NEH. We assessed three strategies including direct 3D classification(3D-CNN); Multiple Instance Learning (MIL), and the Volume-Level Feature Aggregation Transformer (VLFAT). We compared state-of-the-art backbones such as RETFound, MedNet3D, and Vision Transformers (ViT), alongside our proposed FlexiVarViT architecture. We also examined and compared the benefit of OCT-specific pretraining by integrating RETFound weights into our architecture and by performing supervised pretraining on the Kermany dataset.

VLFAT consistently demonstrated superior performance over both MIL and 3D-CNN approaches across all datasets, demonstrating greater robustness and generalization capacity for multi-disease OCT classification, as well as more effective handling of variable-length volumes (i.e., varying numbers of B-scans). In contrast, 3D-CNN models (MedNet3D-R18 and R50) appeared less suitable for this complex task. Their relatively low number of parameters limits their capacity to capture intricate features, and their architecture is poorly adapted to handle variable-length volumes or multi-label classification problems. Additionally, their performance dropped sharply on monoslice datasets like OCTDL, indicating a high sensitivity to the shift from full-volume to single-slice inputs.

MIL-based approaches, although more effective than 3D-CNNs, remained inferior to VLFAT in terms of performance. This difference mainly results from their aggregation and labeling strategies. MIL aggregates predictions at the output level and applies strong labels to each individual slice, assuming that all slices in a volume reflect the same pathology. This can introduce inconsistencies, particularly when pathological signs are localized and not present in every slice. In contrast, VLFAT aggregates features across slices and incorporates spatial position encoding through an attention mechanism, which allows the model to consider the relative position of each slice within the volume. By assigning a single label at the volume level and learning to weight slice-level features accordingly, VLFAT achieves a more coherent and context-aware representation of the full OCT volume, resulting in improved classification accuracy.

All methods experienced a significant drop in performance on the OCTDL dataset. This can be attributed to a domain shift, including differences in acquisition protocols, image resolution, and class distributions between the training set (OCTBrest) and the test set (OCTDL). In addition, OCTDL contains only a single central slice per volume, which limits the structural context available for accurate diagnosis. Another important factor is the labeling strategy: OCTDL uses a multi-class labeling scheme, where each slice is assigned only one pathology, in contrast to the multi-label setup used during training, where multiple co-occurring conditions could be present in a single volume. This inconsistency in labeling paradigms can lead to an underestimation of model performance, especially in cases where multiple pathologies may be present but only a single condition is annotated.

RETFound consistently delivered strong performance, particularly on the“OTHER”class, Likely due to its exposure to a wide range of pathologies during pretraining on 736,000 OCT B-scans. However, incorporating RETFound weights into FlexiVarViT did not lead to notable performance improvements. This suggests that RETFound already provides robust OCT feature representations, limiting the added value of architectural enhancements such as high-resolution processing.

FlexiVarViT outperformed the standard ViT in all settings, especially in out-of-domain scenarios, thanks to its ability to process native-resolution and variable-sized OCT data. Standard models such as ViT require resizing to 224×224, leading to information loss. In contrast, FlexiVarViT dynamically adjusts patch sizes to preserve fine anatomical structures.

FlexiVarViT pretrained on the Kermany dataset significantly outperformed all other models, including those using ImageNet or RETFound weights (p < 0.008). This confirms the value of architecture-specific, domain-specific pretraining for OCT classification. Supervised multi-class pretraining on Kermany likely helped the model to learn pathology-specific features more effectively than masked autoencoding (MAE). On NEH, our pretraining improved overall performance, but led to a slight drop in F1-score and sensitivity, potentially due to overfitting caused by class overlap between Kermany and NEH. Expanding the pretraining dataset to include more rare or underrepresented conditions may help overcome this limitation and further improve performance and generalization.

Finally, in terms of computational cost, FlexiVarViT+VLFAT provided the best trade-off between efficiency and performance. Our current pipeline relies on an ensemble of four models to improve robustness to class imbalance, increasing inference time and memory usage by a factor of four. Future development will focus on optimizing a single high-performing model to reduce resource usage and support real-time clinical deployment.

A complete overview of our final classification framework is illustrated in Figure 1e.

## Limitations

Despite the promising results, this study has several limitations. First, rare pathologies were underrepresented and grouped into a single heterogeneous“OTHER”category, which includes a wide range of infrequent and diverse conditions. This grouping limits the ability to accurately evaluate the model’s performance on specific rare diseases and may mask weaknesses related to underrepresented classes. Second, co-occurring pathologies were not assessed in the external test sets. External validation using OCT datasets with multi-pathology annotations is therefore necessary to better evaluate the model’s performance under realistic clinical conditions. Finally, interpretability was not addressed in this study, despite being essential for clinical adoption and integration into diagnostic workflows.

Future work will aim to overcome these limitations by expanding the dataset to include a broader spectrum of rare and co-occurring pathologies, and by developing interpretability tools. We propose an interpretability strategy that identifies the most relevant slice within each OCT volume and highlights the lesion regions within that slice. This would improve transparency, facilitate clinical understanding of model decisions, and strengthen trust in automated diagnostic systems.

## Conclusion

In this study, we introduced and evaluated a high-resolution transformer-based pipeline for multi-disease OCT classification. Through extensive comparison of deep learning architectures and pretraining strategies, we showed that FlexiVarViT+VLFAT pretrained on Kermany dataset using supervised learning consistently achieves the highest performance across datasets.

Our results confirm the importance of native-resolution processing and volume-level attention-based aggregation for accurate diagnosis. Furthermore, supervised pretraining on high-resolution OCT data offers significant gains over generic large-scale pretraining such as RETFound.

While designed for OCT, our framework is adaptable to other 3D medical imaging modalities such as brain MRI or chest CT, where both resolution and slice count vary. Its flexibility and high-resolution processing make it a strong candidate for generalization across clinical imaging domains.

In summary, this work highlights the importance of matching model design and pretraining to medical imaging characteristics and provides a promising foundation for developing scalable, interpretable, and clinically applicable AI tools.

## Supplementary Information

Supplementary Information 1.

Supplementary Information 2.

## Data Availability

The OCTBrest dataset is not publicly available due to project privacy but can be obtained from the corresponding author upon reasonable request. The OCTDL dataset is publicly available at https://data.mendeley.com/datasets/sncdhf53xc/4. The NEH dataset is publicly available at https://hrabbani.site123.me/available-datasets/dataset-for-oct-classification-50-normal-48-amd-50-dme. The Kermany dataset is publicly available at https://data.mendeley.com/datasets/rscbjbr9sj/3.

## Abbreviations

AI: Artificial intelligence
AMD: Age-related macular degeneration
AUC: Area under the ROC curve
DME: Diabetic macular edema
DL: Deep learning
MIL: Multi-Instance learning
NEH: Noor eye hospital dataset
OCT: Optical coherence tomography
OCTDL: Optical coherence tomography dataset for image-based deep learning methods dataset
OCTIP: Optical coherence tomography image preprocessing
ROC: Receiver operating characteristic
VID: Vitreomacular interface disease
ViT: Vision transformer
VLFAT: Variable length volume feature aggregation
