# Supplementary figures and images for: A robust deep learning classifier for screening multiple retinal diseases on optical coherence tomography
Source: Sci Rep. 2025 Oct 9;15:35334. doi: 10.1038/s41598-025-19286-y (PMC12511338; doi:10.1038/s41598-025-19286-y)

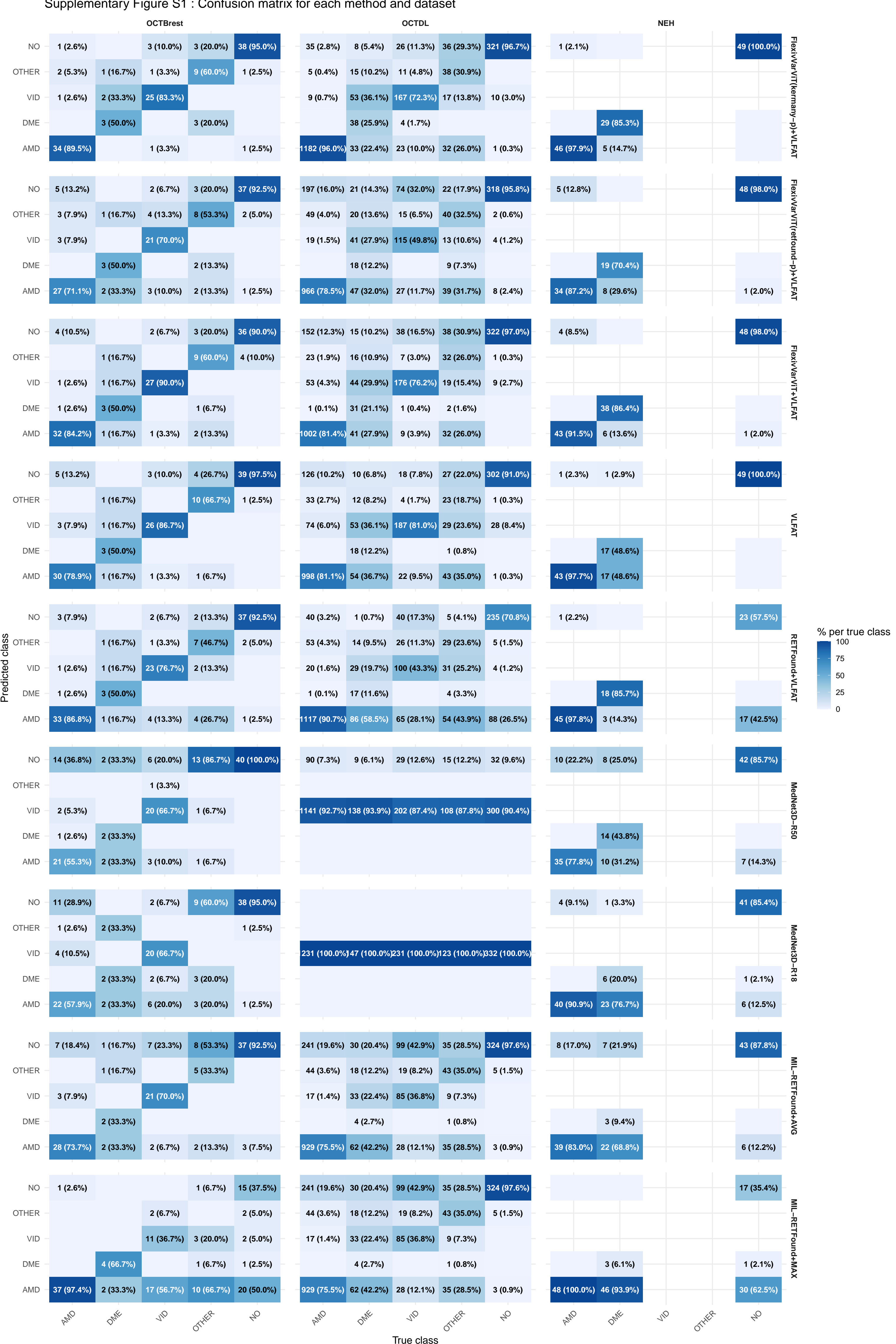

Supplement: Supplementary file 1 — Supplementary Information 1. [file 41598_2025_19286_MOESM1_ESM.pdf]
