# Supplementary material for: A robust deep learning classifier for screening multiple retinal diseases on optical coherence tomography
Source: Sci Rep. 2025 Oct 9;15:35334. doi: 10.1038/s41598-025-19286-y (PMC12511338; doi:10.1038/s41598-025-19286-y)
Supplement: Supplementary file 2 — Supplementary Information 2. [file 41598_2025_19286_MOESM2_ESM.pdf]

Supplementary Table S1 : Class distribution per fold in the OCTBrest dataset

Fold	AMD	DME	VID	OTHER	Total Pathological	Normal	Total	Patient
1	57	8	43	26	98	37	135	51
2	64	5	35	22	93	43	136	50
3	44	8	30	25	81	44	125	50
4	55	8	28	32	97	41	138	50
5	38	6	46	30	89	40	129	50
Total	258	35	182	135	458	205	663	251
